# *PTGDR2* Expression in Peripheral Blood as a Potential Biomarker in Adult Patients with Asthma

**DOI:** 10.3390/jpm11090827

**Published:** 2021-08-24

**Authors:** Asunción García-Sánchez, Miguel Estravís, Maria J. Martin, Jacqueline Pérez-Pazos, Cristina Martín-García, María Gil-Melcón, Jacinto Ramos-González, Ibon Eguiluz-Gracia, Juan Carlos Triviño, María Isidoro-García, Ignacio Dávila, Catalina Sanz

**Affiliations:** 1 Grupo Alergología, Instituto de Investigación Biomédica de Salamanca (IBSAL), 37007 Salamanca, Spain; chonela@usal.es (A.G.-S.); estravis@usal.es (M.E.); mjmartinma@saludcastillayleon.es (M.J.M.); jperezpaz.ibsal@saludcastillayleon.es (J.P.-P.); misidoro@saludcastillayleon.es (M.I.-G.); catsof@usal.es (C.S.); 2 Red Cooperativa de Investigación en Salud–RETICS ARADyAL, 37007 Salamanca, Spain; iboneguiluz@gmail.com; 3 Departamento de Ciencias Biomédicas y del Diagnóstico, Universidad de Salamanca, 37007 Salamanca, Spain; 4 Departamento de Bioquímica y Biología Molecular, Universidad de Salamanca, 37007 Salamanca, Spain; 5 Servicio de Inmunoalergia, Complejo Asistencial Universitario de Salamanca, 37007 Salamanca, Spain; cmartingarci@saludcastillayleon.es; 6 Servicio de Otorrinolaringología, Complejo Asistencial Universitario de Salamanca, 37007 Salamanca, Spain; mgilmel@sacylcastillayleon.es; 7 Servicio de Neumología, Complejo Asistencial Universitario de Salamanca, 37007 Salamanca, Spain; jramosg@sacylcastillayleon.es; 8 Servicio de Alergia, Hospital Regional Universitario de Málaga, 29010 Málaga, Spain; 9 Grupo Bioinformática, Sistemas Genómicos, 46980 Paterna, Spain; jc.trivino@sistemasgenomicos.com; 10 Servicio de Bioquímica Clínica, Complejo Asistencial Universitario de Salamanca, 37007 Salamanca, Spain; 11 Departamento de Medicina, Universidad de Salamanca, 37007 Salamanca, Spain; 12 Departamento de Microbiología y Genética, Universidad de Salamanca, 37007 Salamanca, Spain

**Keywords:** aspirin exacerbated respiratory disease (AERD), asthma, biomarker, chronic rhinosinusitis with nasal polyps (CRSwNP), gene expression, *PTGDR2*

## Abstract

Background: Precision medicine is a promising strategy to identify biomarkers, stratify asthmatic patients according to different endotypes, and match them with the appropriate therapy. This proof-of-concept study aimed to investigate whether gene expression in peripheral blood could provide a valuable noninvasive approach for the molecular phenotyping of asthma. Methods: We performed whole-transcriptome RNA sequencing on peripheral blood of 30 non-atopic non-asthmatic controls and 30 asthmatic patients. A quantitative PCR (qPCR) validation study of *PTGDR2* that encodes for CRTH2 receptor, expressed in cells involved in T2 inflammation, was developed in a cohort of 361 independent subjects: 94 non-asthmatic non-atopic controls, 187 asthmatic patients [including 82 with chronic rhinosinusitis with nasal polyposis (CRSwNP) and 24 with aspirin-exacerbated respiratory disease (AERD)], 52 with allergic rhinitis, and 28 with CRSwNP without asthma. Results: *PTGDR2* was one of the most differentially overexpressed genes in asthmatic patients’ peripheral blood (*p*-value 2.64 × 10^6^). These results were confirmed by qPCR in the validation study, where *PTGDR2* transcripts were significantly upregulated in asthmatic patients (*p* < 0.001). This upregulation was mainly detected in some subgroups such as allergic asthma, asthma with CRSwNP, AERD, eosinophilic asthma, and severe persistent asthma. *PTGDR2* expression was detected in different blood cell types, and its correlation with eosinophil counts showed differences in some groups of asthmatic patients. Conclusions: We found that *PTGDR2* expression levels could identify asthma patients, introduce a minimally invasive biomarker for adult asthma molecular phenotyping, and add additional information to blood eosinophils. Although further studies are required, analyzing *PTGDR2* expression levels in peripheral blood of asthmatics might assist in selecting patients for treatment with specific antagonists.

## 1. Introduction

Asthma is a heterogeneous disease, characterized by chronic airway inflammation with variable respiratory symptoms, affecting around 358 million people worldwide [1,2]. Type 2-driven asthma is a subtype of asthma in which there is a notable release of interleukin-4 (IL4), IL5, and IL13 from cells of both innate and adaptive immune systems [3,4]. These cytokines or their receptors are targets of biological treatments of asthma type 2 [5].

In the precision medicine era, it is necessary to identify inter-individual differences in response to drugs to better stratifying patients finding the most effective treatments for each patient [6]. In this regard, it would be desirable to have specific biomarkers easily measurable in biological fluids (such as blood), minimally invasive, and reproducible. The development of biomarkers that enable clinicians to distinguish phenotypes, identify treatment responders and non-responders, and predict future clinical or adverse outcomes is an unmet need in asthma [7]. Non-coding variants associated with gene expression regulation or epigenetic markers regulating gene expression will yield essential insights into the genetic architecture of asthma [8]. Changes in gene expression constitute the central link between genetic variants, epigenetics, and disease. In this sense, genome-wide assessment of gene expression (transcriptomics) might help identify disease-associated genes and novel disease pathways. Recently, a review of transcriptomic studies in asthma was performed to summarize recent transcriptome studies focusing on asthma pathogenesis and asthma drug responses [9]. As described, blood cells express approximately 80% of the genes encoded by the human genome, and blood contains many of the cells involved in immune responses. In addition, blood is an easily accessible tissue. For these reasons, blood cells have been widely used for asthma transcriptome studies [9]. Some genomic control signatures were described in a transcriptomic study performed in 1170 adult asthmatics with varying degrees of asthma control status [10]. Yeh et al. performed a cluster analysis of gene expression in PBMCs from 133 childhood asthmatics and identified three molecular clusters with distinct inflammatory profiles. Among them, cluster 3 was characterized by changes in glucocorticoid signaling and activation of the Th1/Th17 immune pathways and was related to poor treatment control [11]. Some whole-genome expression studies in blood cells have analyzed the effect of asthma severity [12,13]. In another transcriptomic study, the samples of PBMCs from 118 adult asthmatics were analyzed between the stable and exacerbation states, showing a distinct exacerbation-associated gene expression signature [14]. Finally, the effect of the biological treatment benralizumab on the whole blood transcriptomic signature of severe asthmatic patients was analyzed, showing significant reductions in the expression of genes associated with eosinophilic inflammatory responses [15]. 

In summary, different transcriptomic studies in asthma are adding information on novel genes and related pathways involved in the numerous pathophysiological mechanisms of this heterogeneous disease. In this work, we performed a transcriptomic analysis in whole blood samples of patients with allergic asthma and non-allergic non-asthmatic controls. The study aimed to find differentially expressed genes and analyze whether the determination of any of these gene expression levels could be employed as a biomarker to provide additional information for the phenotypic characterization of patients with asthma.

## 2. Materials and Methods

### 2.1. Study Population

A total of 421 subjects participated in the study, including 60 subjects who participated in the transcriptomic analysis, and 94 controls and 267 patients involved in the validation study. In the transcriptomic analysis, 30 patients with allergic asthma diagnosed by an allergist, and 30 non-asthmatic non-atopic controls were included. For the validation analysis, 361 Caucasian individuals were recruited from the Allergy Department of the Salamanca University Hospital. The Clinical Research Ethics Committee of the Institute for Biomedical Research of Salamanca (IBSAL) approved the study (PI 2020-02-433) and informed written consent from study subjects was obtained. Ninety-five individuals were enrolled as controls. Controls fulfilled the following criteria: (i) no symptoms or history of fulfilled the following criteria: (i) no symptoms or history of asthma or other pulmonary diseases, (ii) no symptoms or history of rhinitis, (iii) no symptoms or history of allergic diseases, (iv) negative skin prick tests to a battery of common aeroallergens; (v) absence of a family history of asthma, rhinitis, or atopy, and (vi) age > 16 years old. Age was significantly higher in controls than in patients in order to permit a more extended period for asthma to have appeared. In addition, the patients were recruited meeting the following criteria: (i) physician-diagnosis of respiratory allergy (asthma, rhinitis, nasal polyposis or aspirin-exacerbated respiratory disease (AERD), (ii) age > 16 years. Skin prick tests were performed from a battery of common aeroallergens previously described [16]. Skin tests were considered positive if there were at least one wheal reaction of >3 mm of diameter bigger than the negative control. The severity of asthma was evaluated according to the Spanish Guide for the Management of Asthma (GEMA 4.4) [17], allergic rhinitis was classified following ARIA guidelines modified by Valero et al. [18]. None was receiving oral corticosteroids; however, asthmatic patients received inhaled corticosteroids therapy.

### 2.2. Transcriptomic RNA Sequencing (RNAseq) and Data Analysis

Total RNA was isolated from peripheral blood samples stored in RNA later at −20 °C using the Ambion RiboPureTM Blood kit (Thermo Fisher Scientific, Waltham, MA, USA). Peripheral blood was selected due to its accessibility. RNA was treated with Ambion DNAse I and concentrated with the RNeasy MinElute Cleanup Kit (Qiagen, Hilden, Germany). All purification protocols were carried out following the manufacturer’s instructions. RNA was quantified with a Nanodrop 1000 instrument, and the RNA quality was assessed by measuring the RIN (RNA Integrity Number) in a Bioanalyzer Agilent 2100 expert equipment using the Eukaryote Total RNA Nano kit (Agilent Technologies, Santa Clara, CA, USA). 1 µg of RNA samples with an RIN higher than 8.0 was used for RNAseq. Globin transcripts and ribosomal RNA were removed, and RNA was cleaved to prepare RNA strand-specific libraries, generate clusters from each library, and perform a high-throughput sequencing using the Illumina HiSeq 2500 ultra-sequencing platform (Illumina, San Diego, CA, USA).

### 2.3. Sequences Selection and Characterization

In the bioinformatic analysis of the transcriptomic obtained data, we include quality control of the raw data using the FastQC tool (http://www.bioinformatics.babraham.ac.uk/projects/fastqc (accessed on December 2019)). The reads were then mapped against the human genome version hg39 using the tophat2 algorithm [19]. The low-quality reads were removed using Picard Tools software (https://broadinstitute.github.io/picard/ (accessed on December 2019)). The unmapped and non-properly paired reads were re-mapped using the bwa mem algorithm [20]. Aligning sequence reads, clone sequences, and assembly contigs were carried out with Burrows–Wheeler Aligner (BWA-MEM). The genes and isoforms predictions were estimated using the Cufflinks method [21] and expression levels were calculated using the HT Seq software version 0.6.0 (http://www-huber.embl.de/users/anders/HTSeq/ (accessed on December 2019)). The differential gene expression between different conditions was identified using the DESeq2 method [22]. We consider as true differently expressed genes those with a fold-change value equal or below −1.5 or equal or higher than 1.5 and with a *p*-value (Padj) corrected by FDR (false discovery rate) <0.05 [23]. Given the heterogeneity of asthma, the role of different clinical and biological markers was included in the bioinformatics study with the purpose of detecting their potential influence in the study. Quantitative PCR (qPCR) was performed on selected genes to validate the sequencing data.

### 2.4. Quantitative PCR Expression Analysis

Total RNA was isolated from peripheral blood samples stored with RNA later at −20 °C, using the RiboPure-Blood kit (Ambion, Thermo Fisher Scientific, Waltham, MA, USA) according to the manufacturer’s instructions. DNAse treatment was performed using Turbo DNAse (Ambion, Thermo Fisher Scientific, Waltham, MA, USA). Concentrations and RNA quality ratios were measured using a NanoDrop 1000 (Thermo Fisher Scientific, Waltham, MA, USA). Reverse transcription (RT) was performed on 500 ng of total RNA with Superscript III First-Strand Synthesis System for RT-PCR (Invitrogen, Thermo Fisher Scientific, Waltham, MA, USA) using a thermal cycler (MultiGene Opti-Max, Labnet International Inc., Edison, NJ, USA) in a total volume of 20 μL, with a single cycle and incubation periods of 65 °C for 5 min, 25 °C for 10 min, 50 °C for 50 min, 85 °C for 5 min, and 37 °C for 20 min. All the analyzed samples were transcribed with the same reverse transcription reaction conditions. Relative qPCR was performed using the LightCycler480^®^ Instrument and SYBR Green I Master (Roche, Basel, Switzerland). Fold induction was calculated using the formula 2-(ΔΔCt) by the comparative Ct method [24].

In a pilot study, we performed the qPCR analysis with both *GAPDH* and *TBP* as reference genes, showing similar *PTGDR1* relative expressions and a high correlation among them (Spearman ρ, 0.783; *p* < 0.001) [16]. Based on this study, we decided to complete the validation study in the broad population with *GAPDH* due to its wide use in the literature. Oligonucleotides targeting *PTGDR2* were designed using the primer analysis software Primer 3.0 [25] and assisted using Beacon Designer Software [26]. *GAPDH* reference gene primers were chosen from the panel Real-Time ready Human Reference Gene (Roche Applied Science, Indianapolis, IN, USA). Primers were used at 400 nM each and cDNA at 20 ng in 15 µL reactions. Conditions for PCR included 10 min at 95 °C followed by 45 cycles of real-time PCR with 3 segments amplification, including 10 s at 95 °C for denaturation, 10 s at 60 °C for annealing, and 10 s at 72 °C for polymerization. The dissociation protocol to determine the melting curve from 60 °C to 95 °C for each PCR product was added after thermocycling to verify that each primer pair produced only a single product. All samples showed only one melting peak, indicating that PCR generated only one amplicon and no primer/dimer formation. The qPCR efficiencies were performed by amplifying a standardized dilution series of the template cDNA and were determined for each gene based on the standard curve slope. The qPCR efficiencies were calculated with the equation: E = (10^−1^/slope^−1^) × 100, obtaining efficiencies values between 90% and 102%. Each sample was performed in triplicate, with non-template controls and calibrators included in each experiment. *PTGDR2* was measured twice over time in several subjects to rule out the intra-patient variability (data not shown). All procedures were performed following MIQE guidelines [27].

### 2.5. Clinical Measurements

Cell peripheral blood was counted automatically using a counter (Beckman Coulter, Brea, CA, USA) and the MAXM A/L system (Beckman Coulter, Brea, CA, USA). Blood cell counts of the transcriptomic study participants are summarized in supplementary data (Table S1). Total IgE serum levels were measured by a fluoroenzyme immunoassay (ImmunoCap System, ThemoFisher Scientific, Waltham, MA, USA). FeNO was determined using NIOX VERO (Circassia, Uppsala, Sweden).

### 2.6. Flow Cytometry

Fresh blood samples from 12 patients were processed and analyzed on the same day. Red blood cells were lysed using lysis buffer (150 mM NH_4_Cl, 12 mM NaHCO_3_, 0.1 mM EDTA). PBMCs were washed twice with PBS previously to incubation with antibodies. Labeling was performed using the following antibodies: CD3-PE, CD123-APC, and CD294-FITC were purchased from BD Biosciences (Franklin Lakes, NJ, USA); CD45-PerCP-Cy5.5 and HLA-DR PB were from Biolegend (San Diego, CA, USA). A FACSAria instrument (BD Biosciences) was used. Samples were analyzed using the Flowing Software from Turku Biosciences (Turku, Finland).

### 2.7. Statistical Analysis

Data analysis was performed using pairwise comparison by analysis of variance (ANOVA), unpaired one-sample *t*-test, Kruskal–Wallis, Pearson’s correlation coefficient, χ^2^ Test of independence, Logistic Regression, and The Receiver Operating Characteristic (ROC) analysis using the SPSS Software, version 26 (IBM, Armonk, NY, USA). Comparisons between ROC curves were performed using MedCal version 19 (MedCalc Software Ltd., Ostend, Belgium). Graphs were plotted using GraphPad Prism version 6 (San Diego, CA, USA). Data were representative of at least three independent experiments. A *p* < 0.05 was considered significant. Statistical controls were applied for statistically significant associations: the analysis of multivariate logistic regression adjusted for potential confounding variables such as age, sex, IgE, and white blood cells counts, using the WALD test; the statistical power (SP) to evaluate the sample size and the false positive report probability (FPRP) using the method to identify the potential type I error [28]. Bonferroni corrections were applied when multiple comparisons are performed considering the original *p*-value as *p* < 0.05 to calculate the new *p*-values.

## 3. Results

### 3.1. Characteristics of the Population of the Transcriptomic Assay

The phenotypic characteristics of the study population are shown in Table 1, clinical information of asthmatics, such as medications, atopic status, smoking status, and co-morbid diseases are described in supplementary data (Table S2). Total IgE levels were significantly lower in controls (*p* < 0.001). All patients had allergic asthma and were sensitized to pollen. Moderate persistent asthma was predominant (46.4%), followed by intermittent asthma (35.7%). Persistent moderate rhinitis (82.8%) and mild intermittent rhinitis (10.3%) were the most frequent types.

### 3.2. Transcriptomic Study

We observed transcriptional differences between asthmatic patients and controls. A search for the most differentially expressed genes was performed, and the top 20 genes with a fold-change value equal to or below −1.5 or equal to or higher than 1.5 and a *p*-value corrected by FDR (Padj) <0.05 were selected. Ten of these genes were upregulated and ten downregulated in patients (Table 2). The Gene Ontology (GO) term analyses for biological processes of the most differentially expressed genes are listed in Table 3. We performed a multivariate logistic regression analysis to adjust the differential expressed genes to the white blood cell counts as covariates. It is described in supplementary data (Table S3).

We selected *PTGDR2* for several reasons: it was one of the five most differentially overexpressed coding transcripts in asthmatic patients (fold change 1.989; *p*-value 2.64 × 10^6^) (Table 2) the role of PGD2 and their receptors, PTGDR1 and PTGDR2, in asthma and allergy [16,29,30,31], and mainly because there are several drugs directed against the receptor in development [32]. Moreover, *PTGDR2* remains differentially expressed after adjusting expression levels through logistic regression with blood cell counts. (Table S3).

**Figure 1:**
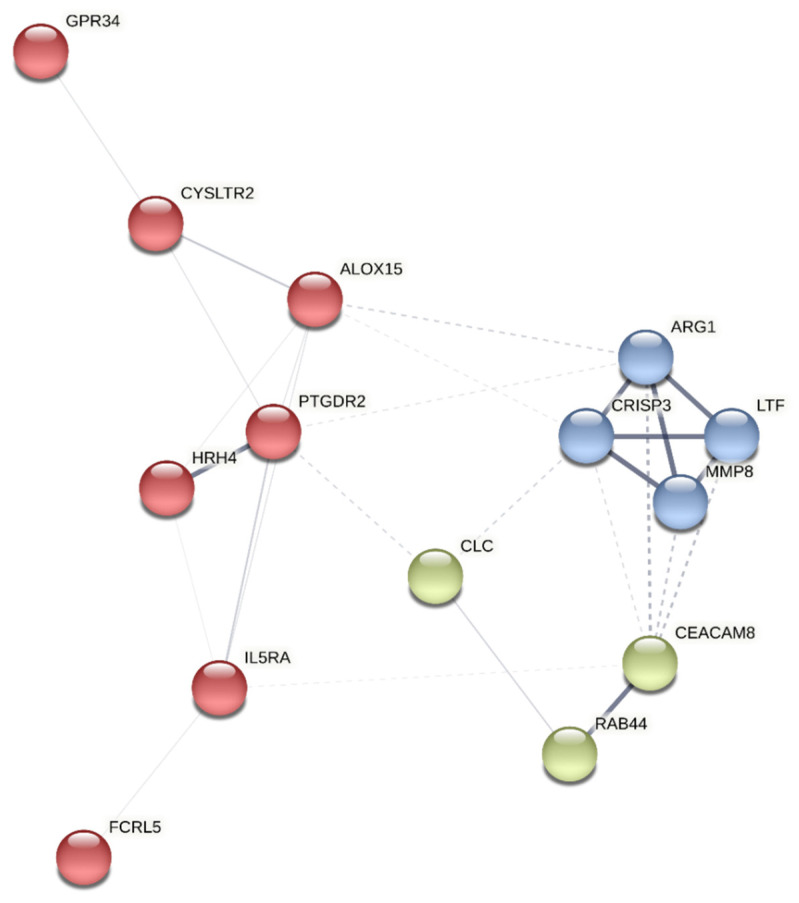
Cluster analysis of the most differentially expressed genes using the STRING platform. The identified clusters are colored in red (A), blue (B), and green (C). The solid and the dotted lines indicate connections within the same and different clusters, respectively.

A protein-protein interaction network analysis was performed on the selected genes using the K-means [33] clustering method with STRING (Search Tool for the Retrieval of Interacting Genes/Protein) [34] to identify clusters within them. The approach yields three clusters: A, B, and C (Figure 1). PTGDR2 was found in cluster A, enriched in proteins involved in immunological processes such as HRH4, ALOX15, CYSLTR2, GPR34, IL5Rα, and FCRL5 (Figure 1) (Table 3).

### 3.3. Characteristics of the Population in the Validation Study

Next, we verified the differential expression of *PTGDR2* observed in the RNAseq assay with a qPCR analysis in a large cohort study. The characteristics of the population of the validation study are shown in Table 4 and Table 5. Patients were diagnosed with atopic or non-atopic asthma, associated or not with CRSwNP. We also included a group of patients who suffered from CRSwNP but that did not have asthma. Only in this group was there a male predominance, although with no significant differences. We also included a group of 52 patients with allergic rhinitis without asthma. Total IgE levels were significantly lower in controls than all subgroups of patients (*p* < 0.001 to *p* < 0.05), except for the group of patients with CRSwNP without atopy. The mean values of blood eosinophilia differed significantly between patients and controls (*p* < 0.001 to *p* < 0.01) except for the group of non-atopic asthma without CRSwNP (Table 5). Concerning asthma severity, moderate persistent asthma was the most common (42.3%), followed by intermittent asthma (25.8%) and mild persistent and severe persistent asthma (15.9% each one). One hundred and twenty-four patients were diagnosed with allergic asthma and allergic rhinitis. 

### 3.4. qPCR Expression Analysis

**Figure 2:**
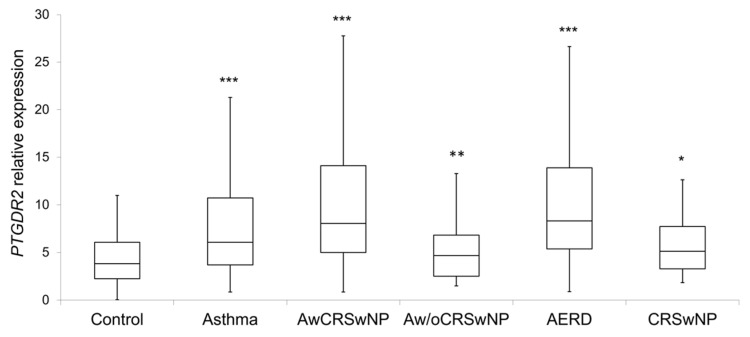
Boxplot of the *PTGDR2* expression levels in controls and patients; CRSwNP: Chronic Rhinosinusitis with Nasal Polyposis; AwCRSwNP: Asthma with CRSwNP; Aw/oCRSwNP; Asthma without CRSwNP; AERD: Aspirin-exacerbated respiratory disease; *** *p* < 0.001; ** *p* < 0.01; * *p* < 0.05; *p*-value of the Kruskal–Wallis test for each group of patient vs. controls after age and sex adjustment.

*PTGDR2* expression levels were significantly higher in patients (8.2 ± 8.3; *p* < 0.001) than in controls (4.4 ± 2.7) (Table 5 and Figure 2). Within the different subgroups, the higher expression was observed in atopic and non-atopic asthmatics with CRSwNP (11.1 ± 10.8, *p* < 0.001; and 10.6 ± 7.8, *p* < 0.001, respectively), AERD (11.7 ± 11.9; *p* < 0.001) (Table 5 and Figure 2), eosinophilic asthma (9.5 ± 7.2; *p* < 0.001), and severe persistent asthma (12.1 ± 11.2; *p* < 0.001) (Table 6). Importantly, all comparisons were confirmed by logistic regression adjusted by age and sex, IgE and eosinophils, except for non-atopic asthma without CRSwNP, CRSwNP without asthma and AERD.

Besides, no significant differences with the control group were observed in non-atopic asthma without CRSwNP (5.8 ± 5.9) or in atopic CRSwNP without asthma (4.8 ± 2.7) (Table 5). 

A group of 52 patients who only had allergic rhinitis (AR) was included to analyze the relationship of atopy with the expression of *PTGDR2*. There were no significant differences in *PTGDR2* expression levels between these 52 AR patients (7.7 ± 11.3) and the control group (4.4 ± 2.7) (Table 5).

Interestingly, increasing expression levels of *PTGDR2* were observed as comorbidities of the patients heightened. Thus, patients with asthma and CRSwNP presented high values of *PTGDR2*, which were further increased in patients who also presented intolerance to NSAIDs (AERD) (Table 5). In addition, higher *PTGDR2* expression levels were observed as the severity of asthma increased. Thus, statistically significant differences were found between controls and patients with moderate persistent asthma and severe asthma (Table 6). Furthermore, patients with mild persistent asthma had the lowest expression levels of *PTGDR2* (7.3 ± 5.0), while patients with severe asthma showed the highest levels (12.1 ± 11.2). In addition, there were statistically significant differences when comparing severe asthma with the rest of asthma severity types (12.1 ± 11.2 vs. 8.0 ± 5.3; *p* = 0.05). We only have the asthma severity classification of 179 patients. 

**Figure 3:**
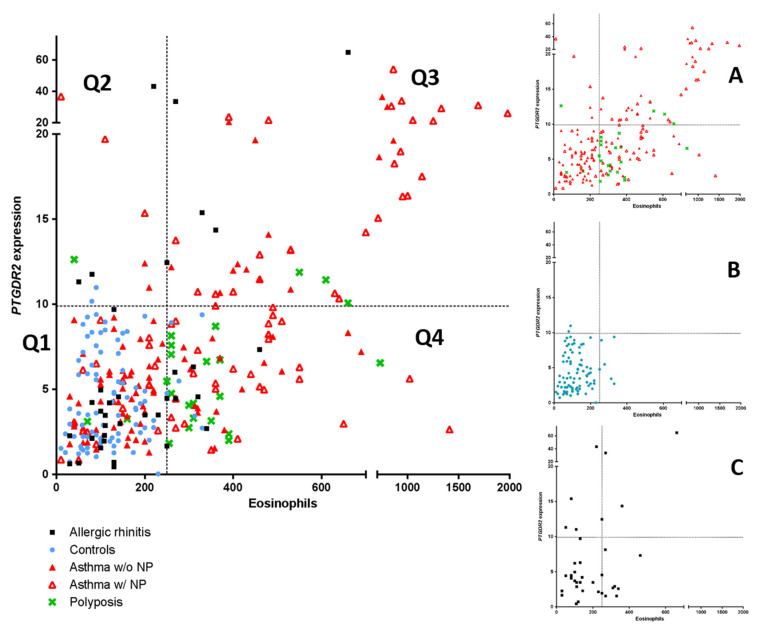
Scatter plot of the linear relationship of *PTGDR2* expression and peripheral eosinophil counts (left panel). Comparison among asthma patients with and without polyposis, simple polyposis, allergic rhinitis, and controls are shown. (Panels **A**–**C**) show the main groups independently. Four quadrants were obtained by dividing according to a positivity cutoff of 250 eosinophils cells/µL (Specificity > 95%) and a cutoff of 9.3-fold difference for *PTGDR2* expression (Specificity >95%): Q1 (EO < 250 cells/µL; *PTGDR2* < 9.4-fold); Q2 (EO < 250 cells/µL; *PTGDR2* > 9.3-fold); Q3 (EO > 250 cells/µL; *PTGDR2* > 94-fold); Q4 (EO > 250 cells/µL; *PTGDR2* < 9.3-fold).

As *PTGDR2* mRNA expression could be a possible biomarker for asthma, a comparison with peripheral blood eosinophil count using an ROC curve analysis was performed. The ROC curve analysis identified blood eosinophil counts as the best global predictor of patients [0.790 (0.741–0.838)] (*p* < 0.05). However, when establishing a positivity cutoff of ≥250 eosinophils peripheral blood eosinophil (specificity ≥ 95%), 46.5% (*n* = 114) of patients of the global sample were erroneously classified as false negatives. In this case, using the *PTGDR2* expression test with a cutoff of 9.3, which also has a specificity of ≥95%, we were able to identify 11 patients (10%) from the total of false negatives. In Figure 3, the mentioned above cutoffs (≥250 eosinophils and ≥9.3 *PTGDR2* expression) determine four quadrants (Q1, Q2, Q3, Q4); these patients were located in the Q2 quadrant and were distributed into the following groups: Rhinitis (4 patients), asthma with polyposis (3 patients, including one with AERD), asthma without polyposis (3 patients) and polyposis without asthma (one patient). This result suggests that the *PTGDR2* expression test is a more specific biomarker in patients with low eosinophil values (<250 eosinophils), giving additional information to eosinophil levels.

In addition, when we performed the ROC curve analysis in the group of asthmatic patients vs non-asthmatic, the area under the curve (AUC) for *PTGDR2* [0.612 (0.533–0.691)] is higher (not significant *p* > 0.05) than for eosinophils [0.587 (0.511–0.663)]. Exploring the CRSwNP patients with or without asthma, we also found a higher (not significant *p* > 0.05) AUG for *PTGDR2* [0.672 (0.566–0.778)] than for eosinophils [0.597 (0.486–0.707)]. Finally, in the case of asthma with CRSwNP, with or without NSAID intolerance respiratory, a higher (not significant *p* > 0.05) AUC was also found for *PTGDR2* than for eosinophils (Table 7).

A moderate correlation (r = 0.587, *p* < 0.01) between *PTGDR2* expression and eosinophil counts was found in the group of asthmatics. However, streaking differences in *PTGDR2* expression were detected in asthmatic patients with similar baseline blood eosinophil levels (Figure 3). Notably, two groups focused our attention, i.e., those with low eosinophil levels and high *PTGDR2* expression (quadrant 2) and those with high eosinophil counts and low *PTGDR2* expression (quadrant 4) (Figure 3, Table 8 and Table 9). This fact was mainly observed in asthmatic patients with CRSwNP and patients with allergic rhinitis (Figure 3). The phenotypic and clinical characteristics of different quadrants are shown in Table 8. Notably, we found significant differences in *PTGDR2* expression among different quadrants using post hoc tests (*p* < 0.001). The distribution among the four quadrants is shown in Table 9. All observed frequencies (control and patient groups) were associated with the corresponding quadrant (*p* < 0.05; using χ^2^ test). It suggests that the combination of the selected cutoffs could efficiently discriminate the patient groups studied. 

### 3.5. Flow Cytometry

**Figure 4:**
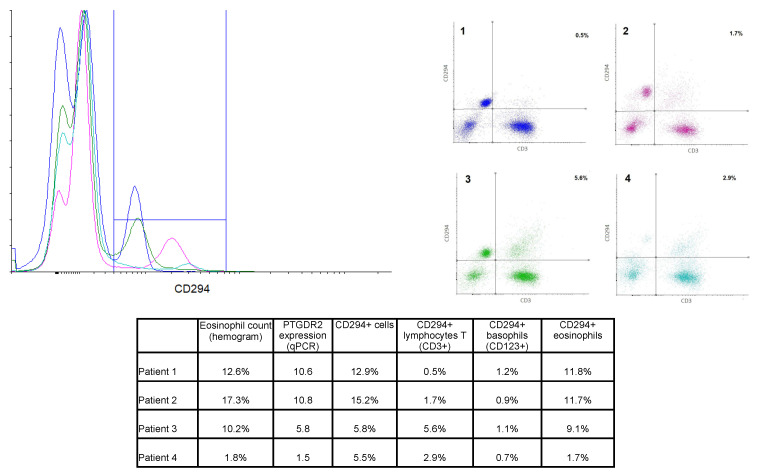
Overlay histogram and dot plots from 4 selected patients. **Left** panel: Overlay histogram showing CD294+ cells in all 4 patients. **Right** panel: Dot plot of CD3-CD294 labeling, percentages of the right upper quadrant (CD3+CD294+ cells) are shown. Color code was kept in both graphs. **Lower** table: Percentage of the different CD294+ cell population in comparison to eosinophil counts and *PTGDR2* expression for the selected four patients.

PTGDR2 PBMCs were labeled using specific antibodies for lymphocytes T (CD45+CD3+) and basophils (CD123+). Eosinophils were identified by their autofluorescence. Cells were also labeled with anti-PTGDR2 (CD294) to determine the protein expression on the cell surface. Results from four representative patients are shown in Figure 4. Patients 2 and 3 were diagnosed with AERD, patient 1 had asthma and bronchospasm with NSAID but not nasal polyposis, and patient 4 had atopic asthma.

Total CD294+ cells ranged from 0.7% to 16% of total cells, and a moderate correlation with *PTGDR2* gene expression was found (R^2^ = 0.6077). Eosinophils were, as expected, the most abundant CD294+ population. Eosinophil count (as per hemogram) was found to correlate with the percentage of CD294+ eosinophil (r^2^ = 0.925, *n* = 12). Thus, patients with the highest eosinophil counts in peripheral blood showed the highest CD294+ labeling on eosinophils (e.g., patients 1 and 2). However, some differences could be appreciated between these two patients. While the eosinophil CD294+ labeling was quite similar (11.8% and 11.7%), total CD294+ labeling differed due to differences in CD3+CD294+ cell percentage, as the level in patient 2 was three-fold that of patient 1 (see table in Figure 4).

Moreover, patients 3 and 4 showed similar levels of CD294+ labeling despite having strikingly different eosinophil counts, most likely due to a greater percentage of CD3+CD294+ cells, i.e., CD294+ T cells.

According to these results, eosinophil counts and other biomarkers, such as *PTGDR2* expression, might provide a more comprehensive view of the role of several cell populations, thus contributing to classifying patients in a more effective, personalized way.

## 4. Discussion

Using whole-transcriptome RNAseq, we explored differentially expressed genes in peripheral blood of asthmatics and healthy individuals. A total of ten significantly upregulated and ten downregulated genes were identified as the topmost differentially expressed. The protein-protein interaction network revealed a cluster of upregulated genes associated with T2 asthma that could further consider acting as asthma biomarkers. *PTGDR2*, one of the most differentially expressed genes, was chosen as a candidate because different antagonists against *PTGDR2* were being developed as a therapy for T2 asthma [35,36,37]. Furthermore, *PTGDR2* expression was a significant predictor variable (*p* < 0.05) when adjusted for white blood cell subtypes (Table S3), which should be taken into account when interpreting heterogeneous samples as blood samples [38]. Then, we underwent the validation study in peripheral blood in a different and broader population of patients. We found statistically significant higher expression levels in patients with asthma, particularly in patients with allergic asthma and AERD as comorbidities. 

Access to high-throughput technologies has made transcriptomic approaches being increasingly applied in the field of allergic diseases. RNAseq technology can thus find transcripts that might become future biomarkers [39]. In the present study, amongst the top 20 transcripts differentially expressed, *PTGDR2* was one of the most differentially overexpressed (Table 2). Interestingly, using the protein-protein interaction network software STRING, PTGDR2 was involved in a network cluster with HRH4, ALOX15, CYSLTR2, GPR34, IL5Rα, and FCRL5 (Figure 1). The genes coding for these proteins were significantly upregulated in our transcriptomic analysis and related to the T2 asthma signature [37,40,41,42,43,44,45,46,47]. In a recent whole blood transcriptomic study of 41 severe asthma patients, in which gene expression levels in peripheral blood were compared at baseline and after four months of treatment with benralizumab [15], significant reductions in the expression of genes associated with eosinophilic inflammatory responses, such as *PTGDR2*, *ALOX15*, *IL5RA*, *SMPD3*, *CLC*, *HRH4*, *CYSLTR2*, and *RAB44*, were observed. That is in agreement with our transcriptional study since these genes were also at the top of our list of genes differentially expressed in asthma. We have recently observed different expression levels of the *IL5RA* gene in asthmatic patients with a possible role in response to benralizumab treatment [48]. *PTGDR2* was also found differentially expressed in a sputum cell transcriptomic expression study of 84 subjects with asthma compared to 27 healthy controls [49]. *PTGDR2* has been related to inflammatory cell activation and recruitment in vitro and in vivo [50,51,52]. Furthermore, PTGDR2+ T cells have been found elevated in nasal mucosa and skin during allergic inflammation in humans [53,54], suggesting a possible utility as a biomarker. In this study, we validated the expression of *PTGDR2* in a larger cohort of asthmatic patients using qPCR (Table 5), showing significantly higher expression levels of *PTGDR2* in patients than in controls, suggesting that *PTGDR2* levels could differentiate asthmatics patients from controls. Besides, we observed significantly higher levels in certain types of asthma, such as atopic and non-atopic asthma. Notably, the highest expression levels were related to more severe and complex asthma (Table 6). These data are in line with Palikhe et al. [55], who found higher levels of *PTGDR2* mRNA in peripheral blood of severe than mild/moderate asthmatics. Increased *PTGDR2* mRNA levels have also been described in the bronchoalveolar lavage of severe asthma patients [56]. We also found that *PTGDR2* was not differently expressed in patients with allergic rhinitis without concomitant asthma, indicating that the levels of *PTGDR2* in peripheral blood might help differentiate allergic patients with and without asthma. A similar result was observed in patients with CRSwNP, in which *PTGDR2* expression levels were only increased in patients with concomitant asthma, suggesting that they could also help differentiate CRSwNP patients with and without asthma. Whether these findings could be helpful in clinical practice or even have a prognostic value (i.e., in the evolution towards asthma) remains unknown. Interestingly, the highest *PTGDR2* levels (54.1-fold change) corresponded to a young male patient with intermittent asthma and CRSwNP who had asthma exacerbations only when aspirin was taken, without symptoms or needed medication out of these episodes. Although it is highly speculative, maybe the high levels of *PTGDR2* expression could suggest a predisposition for evolution to more severe forms of asthma. In any case, these patients should be monitored to evaluate whether the severity of asthma will increase in the future. If this were to occur, the use of *PTGDR2* as a biomarker of asthma severity could identify patients long before they have severe asthma episodes.

There was a moderate positive correlation between *PTGDR2* expression and peripheral blood eosinophils count in most asthma groups (Table 5). Interestingly, there were patients in which results were divergent, suggesting that the expression levels of *PTGDR2* could add relevant information to eosinophil levels. Indeed, as shown in Figure 3, for a similar level of eosinophils, different levels of *PTGDR2* were found, showing significant inter-individual variability. This fact was mainly observed in asthmatic patients with CRSwNP and patients with allergic rhinitis (Figure 3). The highest *PTGDR2* expression in Q2 corresponded with a polysensitized allergic rhinitis patient (43.2-fold), and two asthmatic patients with CRSwNP and sensitivity to NSAIDs (AERD) (36.7- and 19.7-fold, respectively) (Figure 3). On the contrary, the highest eosinophil counts in Q4 corresponded to two asthmatic patients with CRSwNP (140 and 650 cells/µL, respectively). Interestingly, the number of the CRSwNP patients without asthma was higher in Q4 than in other quadrants (73.1%) (Table 9). Whether this fact could add relevant prognostic information remains to be elucidated.

We found that PTGDR2 (CD294) positive lymphocytes should also be considered when studying PTGDR2 expressing cell populations. Although eosinophils were the most abundant CD294+ cells, some patients exhibited higher levels of CD3+CD294+ than others with similar CD294+ eosinophil levels, showing that T cells could also be relevant. Accordingly, Palihke et al. reported that *PTGDR2* expression levels correlated with PTGDR2 mean fluorescent intensity on CD4+CRTH2+ T cells but not with CRTH2+ eosinophils assessed by flow cytometry [55]. The authors also found that severe asthmatics with a high level of *PTGDR2* mRNA had significantly more surface *PTGDR2* in CD4+ T cells compared with those with low levels of *PTGDR2* mRNA, suggesting that the high level of *PTGDR2* mRNA in severe asthmatics could be driven by the CD4+ T cell population [55]. In this sense, we found no significant correlation with eosinophil counts in patients with AERD, who had the highest levels of *PTGDR2* expression. Moreover, CD3+CD294+ cell percentages in some AERD patients (e.g., patient 3) were higher than in others (e.g., patient 2), showing that this cell population could be relevant at least in a group of AERD patients. Also in these patients, levels of PGD2 are high, both basally and during acute reactions to NSAID [57]. PGD2 overproduction occurs secondary to mast cell and eosinophil activation through thromboxane receptors, PTGDR2 and PTGDR, thus stimulating Th2 cells [57]. Therefore, *PTGDR2* mRNA expression levels could be particularly helpful in AERD patients.

In recent years there has been a significant research effort in the field of PTGDR2 antagonists [3,5,40,58]. Although some phase II studies showed promising asthma results [59,60,61], LUSTER-1 and LUSTER-2 phase III trials were discouraging [62], resulting in no further development of fevipiprant for asthma [63]. Two-thirds of the LUSTER trial patients should have blood eosinophil counts of 250 cells/µL or higher at inclusion. Maybe the determination of *PTGDR2* levels could have provided better patient selection and given better results. A clear example is what happened with mepolizumab, which failed in the initial studies due to an inappropriate patient selection [64]. Kerstjens and Gosens [65], commenting about LUSTER results, considered that, despite these negative results, this was not the end of a chapter but the beginning of a new one. A combination of *PTGDR2* mRNA and blood eosinophils count could provide an optimal predictive value for asthma, CRSwNP, and AERD. That might be particularly relevant in those patients with low eosinophils and high *PTGDR2* expression levels.

One of the limitations of the study is that it is a unicentric study. Nevertheless, this fact gives uniformity to the study. Additionally, we have mainly focused on *PTGDR2*, and other genes may also be relevant in response to treatments. Nevertheless, we selected PTGDR2 by its crucial implication in the immunology of type 2-asthma and because different antagonists are directed against this molecule. 

## 5. Conclusions

In the present work, we explored *PTGDR2* levels in peripheral blood as a possible biomarker demonstrating differential expression levels between asthmatic and controls and progressively increasing *PTGDR2* levels as the severity of asthma raised. Finally, we found patients with low eosinophil counts and high *PTGDR2* levels and vice versa, thus providing additional information to peripheral eosinophil counts. Concerning these findings, we propose that peripheral blood mRNA levels of different genes (such *PTGDR2*) should be explored as new minimally invasive biomarkers that might assist in the molecular phenotyping of asthma and selecting patients for treatment with specific antagonists.

## Figures and Tables

**Table 1:** Phenotypic characteristics of the population of the transcriptomic assay.

Characteristic	Patients	Controls	*p* ^§^
Subjects	30	30	
Sex (%)			
Female	43.3	46.7	
Male	56.7	53.3	
Mean ± SD Age (y)	29.9 ± 12.6	56.5 ± 17.1	<0.001
Mean ± SD IgE (kU/L)	264.5 ± 258.3	76.5± 92.5	<0.001
Mean ± SD EO (/µL)	436.7 ± 243.7	186.3 ± 122.7	<0.001

SD, Standard deviation; EO, absolute eosinophils count per microliter; ^§^ *p* value of the Kruskal–Wallis test for the comparison between the patients and control groups after age and sex adjustment. *p* < 0.05 was considered significant.

**Table 2:** Protein-coding transcripts most differentially expressed between patients with allergic asthma and controls. *PTGDR2* gene is highlighted in bold.

**Upregulated Genes**				
**Ensemble ID**	**External ID Gene**	**Fold Difference**	***p* Value**	***p* adj**
ENSG00000161905	*ALOX15*	2.454698669	3.91 × 10^5^	0.003497494
ENSG00000091181	*IL5RA*	2.224932237	8.63 × 10^8^	6.33 × 10^5^
ENSG00000103056	*SMPD3*	2.166999266	1.44 × 10^7^	940 × 10^5^
ENSG00000105205	*CLC*	2.046574167	4.6 3 × 10^6^	0.001025892
**ENSG00000183134**	***PTGDR2***	**1.98857257**	**2.64 × 10^6^**	0.000743526
ENSG00000134489	*HRH4*	1.929424839	7.21 × 10^7^	0.000293744
ENSG00000152207	*CYSLTR2*	1.842833578	1.15 × 10^9^	2.64 × 10^6^
ENSG00000171659	*GPR34*	1.83179816	1.27 × 10^8^	1.94 × 10^5^
ENSG00000198502	*FCRL5*	1.754373528	3.18 × 10^5^	0.003189346
ENSG00000143297	*RAB44*	1.737180256	8.10 × 10^6^	0.001025892
**Downregulated Genes**				
**Ensemble ID**	**External ID Gene**	**Fold Difference**	***p* Value**	***p* adj**
ENSG00000118113	*MMP8*	−2.7537698	0.0003	0.012689748
ENSG00000012223	*LTF*	−2.3543486	0.0005	0.015835504
ENSG00000124469	*CEACAM8*	−2.1934698	0.0025	0.038985829
ENSG00000123689	*G0S2*	−2.0234872	0.0050	0.058344874
ENSG00000118520	*ARG1*	−1.9991881	4.51 × 10^5^	0.003737597
ENSG00000168209	*DDIT4*	−1.9110235	0.0004	0.014644995
ENSG00000179094	*PER1*	−1.8569679	0.0007	0.019560866
ENSG00000235169	*SMIM1*	−1.8507311	0.0485	0.207401788
ENSG00000255823	*MTRNR2L8*	−1.793644	0.0371	0.179397531
ENSG00000096006	*CRISP3*	−1.7836259	0.0020	0.034730148

**Table 3:** Gene ontology biological process enrichment analysis for most differentially expressed genes. *PTGDR2* gene is highlighted in bold.

Term ID	Description	FDR	Genes
GO:0002252	Immune effector process	0.00060	*LTF*, *MMP8*, *CEACAM8*, *CXCL5*, *DDIT4*, *ARG1*, *CRISP3*, *RAB44*
GO:0002376	Immune system process	0.00060	*SMPD3*, *LTF*, *MMP8*, *CEACAM8*, *CYSLTR2*, *CXCL5*, *DDIT4*, ***PTGDR2***, *ARG1*, *CRISP3*, *IL5RA*, *RAB44*
GO:0006955	Immune response	0.00060	*LTF*, *MMP8*, *CEACAM8*, *CYSLTR2*, ***PTGDR2***, *ARG1*, *CRISP3*, *IL5RA*, *RAB44*
GO:0043312	Neutrophil degranulation	0.0013	*LTF*, *MMP8*, *CEACAM8*, *ARG1*, *CRISP3*, *RAB44*
GO:0006952	Defense response	0.0018	*LTF*, *HRH4*, *DDIT4*, *ARG1*, *CRISP3*, *IL5RA*, *ALOX15*
GO:0032940	Secretion by cell	0.0025	*SMPD3*, *LTF*, *MMP8*, *CEACAM8*, *ARG1*, *CRISP3*, *RAB44*
GO:0001817	Regulation of cytokine production	0.0185	*CLC*, *LTF*, *PER1*, *ARG1*, *IL5RA*
GO:0002376	Regulation of T cell cytokine production	0.0198	*CLC*, *ARG1*
GO:0071549	Cellular response to dexamethasone stimulus	0.0198	*DDIT4*, *ARG1*
GO:0002819	Regulation of adaptive immune response	0.0206	*CLC*, *ARG1*, *ALOX15*
GO:0009966	Regulation of signal transduction	0.0206	*LTF*, *HRH4*, *CYSLTR2*, *DDIT4*, *PER1*, ***PTGDR2***, *ARG1*, *G0S2*, *ALOX15*
GO:0046006	Regulation of activated T cell proliferation	0.0276	*CLC*, *ARG1*
GO:0050896	Response to stimulus	0.0280	*LTF*, *MMP8*, *CEACAM8*, *HRH4*, *CYSLTR2*, *DDIT4*, *PER1*, ***PTGDR2***, *ARG1*, *G0S2*, *GPR34*, *CRISP3*, *IL5RA*, *ALOX15*, *RAB44*
GO:0048583	Regulation of response to stimulus	0.0287	*CLC*, *LTF*, *HRH4*, *CYSLTR2*, *DDIT4*, *PER1*, ***PTGDR2***, *ARG1*, *G0S2*, *ALOX15*
GO:0002820	Negative regulation of adaptive immune response	0.0293	*ARG1*, *ALOX15*
GO:0016192	Vesicle-mediated transport	0.0407	*LTF*, *MMP8*, *CEACAM8*, *ARG1*, *CRISP3*, *ALOX15*, *RAB44*
GO:0043901	Negative regulation of multi-organism process	0.0407	*LTF*, ***PTGDR2***, *ARG1*
GO:0006954	Inflammatory response	0.0422	*HRH4*, *CXCL5*, *IL5RA*, *ALOX15*
GO:0032963	Collagen metabolic process	0.0422	*MMP8*, *ARG1*

GO, Gene Ontology; FDR, False Discovery Rate.

**Table 4:** Phenotypic characteristics of the population in the validation study.

	N	Sex ^†^	Age, Year	IgE, kU/L	*p* ^§^
Controls	94	69.1	59.2 ± 17.9	62.3 ± 98.5	
Patients	267	51.3	42.6 ± 19.0	285.2 ± 422.9	<0.001
Allergic Rhinitis	52	55.8	29.8 ± 10.8	210.4 ± 348.2	<0.001
Asthma	187	52.9	44.9 ± 18.9	328.7 ± 458.7	<0.001
Atopic	124	47.6	37.6 ± 16.4	409.7 ± 521.5	<0.001
Non-atopic	63	63.5	59.1 ± 15.1	161.1 ± 207.6	<0.001
A w/CRSwNP	82	46.3	54.4 ± 16.4	310.2 ± 416.7	<0.001
Atopic	43	41.9	46.0 ± 15.8	417.9 ± 508.0	<0.001
Non-atopic	39	51.3	62.6 ± 12.7	184.7 ± 222.9	<0.001
A w/o CRSwNP	105	58.1	37.8 ± 17.7	342.7 ± 489.6	<0.001
Atopic	81	50.6	33.2 ± 15.0	405.5 ± 531.6	<0.001
Non-atopic	24	83.3	53.5 ± 17.3	124.1 ± 179.7	0.05
AERD **	24	45.8	57.2 ± 13.0	304.7 ± 354.8	<0.001
CRSwNP w/o A	28	32.1	50.9 ± 20.7	140.7 ± 208.3	<0.05
Atopic	14	14.3	46.1 ± 18.1	200.2 ± 238.5	<0.001
Non-atopic	14	50.0	55.6 ± 22.6	81.2 ± 160.1	NS

Indicates the number of cases or the mean ± SD; ^†^ Female Sex (percentage); A: asthma; CRSwNP: Chronic Rhinosinusitis with Nasal Polyposis; w/: with; w/o: without; AERD: Aspirin-exacerbated respiratory disease. ** These AERD subjects were included in A w CRSwNP (14 non-atopic and 11 atopic); EO: eosinophils; FeNO: Fraction Exhaled Nitric Oxide; ppb: parts per billion; ^§^ *p*-value of the Kruskal–Wallis (KW)-test for each group of patients vs. controls after age and sex adjustment.

**Table 5:** Clinical characteristics of the population in the validation study.

	EO, Cells/µL	*p* ^§^	FeNO, ppb	*PTGDR2*	*p* ^§^	*p* ^¶^	r
Controls	118.9 ± 75.2		n/a	4.4 ± 2.7			0.390
Patients	329.3 ± 287.3	<0.001	44.3 ± 46.8	8.2 ± 8.3	<0.001	0.004	0.518
Allergic Rhinitis	188.4 ± 139.3	<.01	n/a	7.7 ± 11.3	NS	NS	0.585
Asthma	360.7 ± 316.5	<0.01	45.4 ± 47.0	8.7 ± 7.8	<0.001	0.006	0.587
Atopic	355.7 ± 279.4	<0.001	47.8 ± 48.1	8.6 ± 8.4	<0.001	0.008	0.566
Non-atopic	370.0 ± 378.6	<0.001	40.1 ± 44.7	8.6 ± 7.4	<0.001	0.026	0.648
A w/ CRSwNP	483.2 ± 386.2	<0.001	62.7 ± 68.5	10.8 ± 9.4	<0.001	0.013	0.530
Atopic	483.9 ± 357.5	<0.001	69.3 ± 79.2	11.1 ± 10.8	<0.001	0.013	0.526
Non-atopic	482.4 ± 419.1	<0.001	55.3 ± 55.6	10.6 ± 7.8	<0.001	0.011	0.574
A w/o CRSwNP	252.7 ± 187.6	<0.001	34.8 ± 23.5	7.0 ± 5.8	0.001	0.031	0.612
Atopic	273.4 ± 183.2	<0.001	38.1 ± 23.6	7.3 ± 5.8	<0.001	0.005	0.541
Non-atopic	187.6 ± 190.6	NS	24.0 ± 20.5	5.8 ± 5.9	NS	NS	0.836
AERD **	482.3 ± 295.8	<0.001	30.4 ± 22.5	11.7 ± 11.9	<0.001	NS	0.301
CRSwNP w/o A	328.9 ± 177.7	<0.001	n/a	5.9 ± 3.1	0.028	NS	0.312
Atopic	291.8 ± 183.2	<0.001	n/a	4.8 ± 2.7	NS	NS	−0.112
Non-atopic	360.4 ± 173.8	<0.001	n/a	7.0 ± 3.2	0.005	NS	0.617

Indicates the mean ± SD; A: asthma; CRSwNP: Chronic Rhinosinusitis with Nasal Polyposis; w/: with; w/o: without; AERD: Aspirin-exacerbated respiratory disease. ** These AERD subjects were included in A w CRSwNP (14 non-atopic and 11 atopic); EO: eosinophils; FeNO: Fraction Exhaled Nitric Oxide; ppb: parts per billion; n/a: not available; ^§^ *p*-value of the Kruskal–Wallis (KW)-test for each group of patients vs. controls after age and sex adjustment. ^¶^ *p*-value of the KW-test for each group of patients vs. controls after age, sex, IgE, and eosinophils adjustment; *p* < 0.05 was considered significant; r: Pearson correlation between EO and *PTGDR2*-expression, significance at the 0.01 level in bold.

**Table 6:** Peripheral blood eosinophil, FeNO, and *PTGDR2* expression levels according to asthma severity.

	N	*PTGDR2*	*p* ^†^	FeNO	EO	*p* ^†^
Control	94	4.4 ± 2.7		n/a	118.9 ± 75.2	
Intermittent	47	8.2 ± 9.8	0.11	33.3 ± 25.0	307.2 ± 293.4	0.001
Mild Persistent	29	7.3 ± 5.0	NS	57.3 ± 76.4	341.5 ± 237.9	<0.001
Moderate Persistent	77	8.0 ± 5.3	0.004	46.0 ± 41.4	355.9 ± 302.1	<0.001
Severe Persistent	26	12.1 ± 11.2	0.011	48.4 ± 50.2	406.5 ± 417.6	<0.001

Indicates number of cases or the mean media ± SD; FeNO, Fraction Exhaled Nitric Oxide; ppb: parts per billion; EO, eosinophils cells/µL; ^†^ Post hoc test vs. Control; Kruskal–Wallis test: *p* < 0.001.

**Table 7:** ROC curve analysis in patients.

Patients	AUC (95% CI)	
	*PTGDR2*	Eosinophils
Asthma vs. non asthma	0.612 (0.533–0.691)	0.587 (0.511–0.663)
CRSwNP: Asthma vs. non asthma	0.672 (0.566–0.778)	0.597 (0.486–0.707)
CRSwNP + Ashtma: AERD vs. non AERD	0.503 (0.365–0.641)	0.379 (0.245–0.514)

AUC: Area under the curve; CI: Confidence Interval; CRSwNP: Chronic Rhinosinusitis with Nasal Polyposis; AERD: Aspirin-exacerbated respiratory disease.

**Table 8:** Phenotypic and clinic characteristics of the four quadrants obtained by dividing according to values for eosinophils count (250 cells/µL) and *PTGDR2* expression in controls (9.3-fold difference) both with a specificity ≥ 95%).

**Q1 (EO < 250 Cells/µL; *PTGDR2* < 9.3-Fold Difference)**							
	**N**	**Sex ^†^**	**Age, Year**	**IgE, kU/L**	**FeNO**	**EO**	***PTGDR2***
Controls	81	69.1	60.9 ± 17.0	53.7 ± 85.0	n/a	105.4 ± 59.4	4.1 ± 2.5
Allergic Rhinitis	23	60.9	31.4 ± 10.3	204.9 ± 393.6	n/a	104.8 ± 50.0	2.8 ± 1.4
Asthma	77	58.4	44.2 ± 18.4	200.5 ± 190.0	31.5 ± 26.0	135.5 ± 64.6	4.5 ± 2.2
Atopic	46	54.3	34.8 ± 13.2	253.1 ± 200.48	36.9 ± 29.7	141.1 ± 59.9	4.6 ± 2.1
Non-atopic	31	64.5	57.9 ± 16.3	119.0 ± 140.2	22.2 ± 14.1	127.2 ± 71.3	4.4 ± 2.3
A w/CRSwNP	18	33.3	57.8 ± 12.9	152.7 ± 148.0	51.8 ± 47.4	132.9 ± 77.2	4.6 ± 2.5
A w/o CRSwNP	59	66.1	40.3 ± 18.0	214.8 ± 199.7	26.8 ± 15.1	136.2 ± 61.0	4.5 ± 2.1
CRSwNP w/o A	3	0.0	47.0 ± 5.0	60.5 ± 50.7	n/a	150.0 ± 75.56	3.2 ± 0.1
AERD ^‡^	7	14.3	60.4 ± 10.7	73.5 ± 55.8	33.5 ± 27.1	130.4 ± 76.1	5.1 ± 2.9
**Q2 (EO < 250 Cells/µL; *PTGDR2* ≥ 9.3-Fold Difference)**							
	**N**	**Sex ^†^**	**Age, Year**	**IgE, kU/L**	**FeNO**	**EO**	***PTGDR2***
Controls	3	66.7	68.0 ± 4.0	179.4 ± 257.3	n/a	86.7 ± 5.7	10.2 ± 0.8
Allergic Rhinitis	4	25.0	27.7 ± 14.6	477.7 ± 609.6	n/a	120.0 ± 74.4	19.0 ± 16.6
Asthma	6	50.0	48.3 ± 24.8	376.9 ± 528.6	38.3 ± 31.7	143.3 ± 77.4	17.9 ± 9.7
Atopic	5	40.0	42.4 ± 22.4	450.2 ± 555.9	40.2 ± 35.1	132.0 ± 80.7	18.4 ± 10.8
Non-atopic	1	100.0	78.0	10.6	29.0	200.0	15.3
A w/CRSwNP	3	33.3	63.0 ± 13.7	290.5 ± 452.1	19.0 ± 15.6	106.7 ± 95.0	23.9 ± 11.3
A w/o CRSwNP	3	66.7	33.7 ± 26.5	463.3 ± 686.9	57.7 ± 33.8	180.0 ± 43.6	11.9 ± 0.8
CRSwNP w/o A	1	0.0	25.0	40.0	n/a	40.0	12.6
AERD ^‡^	1	0.0	60.0 ± 0.0	812.0 ± 0.0	n/a	10.0 ± 0.0	36.7 ± 0.0
**Q3 (EO ≥ 250 Cells/µL; *PTGDR2* ≥ 9.3-Fold Difference)**							
	**N**	**Sex ^†^**	**Age, Year**	**IgE, kU/L**	**FeNO**	**EO**	***PTGDR2***
Controls	1	100	77	57.1	n/a	330	9.3
Allergic Rhinitis	6	83.3	25.8 ± 8.2	190.8 ± 168.8	n/a	394.2 ± 156.4	25.3 ± 21.1
Asthma	48	45.8	45.1 ± 20.6	559.2 ± 735.2	62.5 ± 65.0	673.5 ± 363.0	17.9 ± 8.9
Atopic	30	33.3	35.9 ± 17.6	743.5 ± 837.2	67.0 ± 75.1	662.3 ± 316.9	17.9 ± 9.7
Non-atopic	18	66.7	60.6 ± 15.5	225.3 ± 304.5	53.9 ± 41.5	962.2 ± 438.8	18.0 ± 7.4
A w/CRSwNP	31	45.2	52.4 ± 18.8	386.9 ± 535.6	70.3 ± 82.6	752.9 ± 413.0	18.4 ± 9.6
A w/o CRSwNP	17	47.1	31.9 ± 17.1	843.1 ± 930.0	51.4 ± 24.0	582.8 ± 181.4	17.0 ± 7.4
CRSwNP w/o A	3	66.7	71.3 ± 13.2	222.7 ± 352.3	n/a	606.7 ± 55.1	11.1 ± 09
AERD ^‡^	9	55.6	52.8 ± 16.6	452.8 ± 431.1	33.2 ± 27.2	571.1 ± 259.6	18.8 ± 13.8
**Q4 (EO ≥ 250 Cells/µL; *PTGDR2* < 9.3-Fold Difference)**							
	**N**	**Sex ^†^**	**Age, Year**	**IgE, kU/L**	**FeNO**	**EO**	***PTGDR2***
Controls	6	83.3	41.7 ± 17.5	81.6 ± 110.6	n/a	281.7 ± 32.5	5.1 ± 2.2
Allergic Rhinitis	8	37.5	36.6 ± 13.9	205.2 ± 351.4	n/a	308.5 ± 69.7	4.7 ± 1.9
Asthma	47	55.3	46.5 ± 17.8	336.6 ± 362.7	59.9 ± 53.6	438.0 ± 209.7	5.4 ± 2.2
Atopic	35	54.3	42.3 ± 17.4	386.2 ± 395.0	51.5 ± 37.8	407.0 ± 132.8	5.3 ± 2.2
Non-atopic	12	58.3	58.8 ± 12.5	191.6 ± 192.3	91.2 ± 94.3	528.3 ± 342.6	5.9 ± 2.2
A w/CRSwNP	28	57.1	54.2 ± 15.2	340.9 ± 393.2	77.0 ± 67.3	450.0 ± 245.7	5.7 ± 2.2
A w/o CRSwNP	19	52.6	35.2 ± 15.2	330.1 ± 322.9	49.9 ± 43.9	420.4 ± 145.9	5.2 ± 2.3
CRSwNP w/o A	19	36.8	51.6 ± 21.5	119.7 ± 159.1	n/a	339.2 ± 107.2	5.1 ± 2.1
AERD ^‡^	7	71.4	59.4 ± 11.9	261.2 ± 305.6	32.3 ± 10.2	431.4 ± 270.5	5.4 ± 2.0

Indicates number of cases (percentage) or mean media ± SD; ^†^ Female Sex; CRSwNP: Chronic Rhinosinusitis with Nasal polyposis; A w/CRSwNP: Asthma with CRSwNP; A w/o CRSwNP: Asthma without CRSwNP; CRSwNP w/o A: CRSwNP without asthma; AERD: Aspirin exacerbated respiratory disease. ^‡^ These AERD subjects were included in Asthma with NP; EO: absolute eosinophils count (cells/µL); FeNO: Fraction Exhaled Nitric Oxide; ppb: parts per billion; n/a: not available.

**Table 9:** Distribution of controls and patients among the four quadrants obtained by dividing according to values for eosinophil counts (≥250 cells/µL) and *PTGDR2* expression in controls (≥9.3-fold difference), both with a specificity ≥ 95%.

Group	Quadrant								*p* *	Total	
	Q1		Q2		Q3		Q4				
	N	%	N	%	N	%	N	%		N	%
Control	81	89.0	3	3.3	1	1.1	6	6.6	<0.05	91	100
AR	23	56.1	4	9.8	6	14.6	8	19.5	<0.05	41	100
Asthma	77	43.3	6	3.4	48	27.0	47	26.4	<0.05	178	100
AA	46	39.7	5	4.3	30	25.9	35	30.2	<0.05	116	100
NAA	31	50.0	1	1.6	18	29.0	12	19.4	<0.05	62	100
A w/CRSwNP	18	22.5	3	3.8	31	38.8	28	35.0	<0.05	80	100
A w/o CRSwNP	59	60.2	3	3.1	17	17.3	19	19.4	<0.05	98	100
AERD	7	29.2	1	4.2	9	37.5	7	29.2	<0.05	24	100
CRSwNP w/o A	3	11.5	1	3.8	3	11.5	19	73.1	<0.05	26	100

AR: Allergic Rhinitis; CRSwNP: Chronic Rhinosinusitis with Nasal polyposis; A w/CRSwNP: Asthma with CRSwNP; A w/o CRSwNP: Asthma without CRSwNP; CRSwNP w/o A: CRSwNP without asthma; AERD: Aspirin exacerbated respiratory disease. We only have eosinophil counts of 91 controls and 245 patients. * All observed frequencies were significant by independence Test (χ^2^ test) (*p* < 0.05).

## Data Availability

The required data is now publicly accessible in the NCBI repository with the code PRJNA686899.

## Supplementary Materials

The following are available online at https://www.mdpi.com/article/10.3390/jpm11090827/s1, Table S1: White blood cell counts in the population of the transcriptomic analysis, Table S2: Clinical information of the population in the transcriptomic analysis, Table S3: Multivariate logistic regression of the most differentially expressed genes in the transcriptomic analysis after adjustment with white blood cell counts.
