# Supplementary material for: PTGDR2 Expression in Peripheral Blood as a Potential Biomarker in Adult Patients with Asthma
Source: J Pers Med. 2021 Aug 24;11(9):827. doi: 10.3390/jpm11090827 (PMC8468563; doi:10.3390/jpm11090827)
Supplement: Supplementary file 1 [file jpm-11-00827-s001.zip › jpm-1314349-supplementary.pdf]

Supplementary Data

Table S1. White blood cell counts in the population of the transcriptomic analysis.

**Controls (n=30)**	**Cells counts/ μl**					
	**Leu**	**Neu**	**Lymph**	**Mon**	**Eos**	**Bas**
Mean	7676,50	4495,00	2443,00	540,07	154,93	43,10
SD	2917,79	2449,75	1362,82	172,96	102,42	33,26
Min	4200	2140	700	260	0	10
Max	15000	13520	7980	930	410	140
**Patients (n=30)**	**Cells counts/ uL**					
	**Leu**	**Neu**	**Lymph**	**Mon**	**Eos**	**Bas**
Mean	6980,67	3700,00	2321,00	499,63	350,90	45,07
SD	1463,72	1182,09	689,07	115,56	212,86	22,81
Min	4340	1530	1250	320	70	0
Max	9390	6290	4620	840	830	100

Leu: Leukocytes; Neu: Neutrophils; Lymph: Lymphocytes; Mon: Monocytes; Eos: Eosinophils; Bas: Basophils; SD: Standard Deviation; Min: Minimum; Max: Maximum

Table S2. Clinical information of the population in the transcriptomic analysis.

CONTROLS							
Sample (Nº)	Atopy				Medication	Comorbidities	Smoking status
	M	P	E	F			
D1 20	N	N	N	N	N	N	N
D1 32	N	N	N	N	N	N	N
D1 81	N	N	N	N	N	N	N
D2 28	N	N	N	N	N	N	N
D2 63	N	N	N	N	N	N	N
D2 76	N	N	N	N	N	N	N
D3 02	N	N	N	N	N	N	N
D3 13	N	N	N	N	N	N	N
D3 17	N	N	N	N	N	N	N
D3 18	N	N	N	N	N	N	N
D3 40	N	N	N	N	N	N	N
D3 47	N	N	N	N	N	N	N
D3 48	N	N	N	N	N	N	N
D3 53	N	N	N	N	N	N	N
D3 68	N	N	N	N	N	N	N
D3 78	N	N	N	N	N	N	N
D4 05	N	N	N	N	N	N	N
D4 24	N	N	N	N	N	N	N
D4 45	N	N	N	N	N	N	N
D4 71	N	N	N	N	N	N	N
D5 20	N	N	N	N	N	N	N
D5 22	N	N	N	N	N	N	N
D6 04	N	N	N	N	N	N	N
D6 19	N	N	N	N	N	N	N

D6 32	N	N	N	N	N	N	N
D6 44	N	N	N	N	N	N	N
D6 71	N	N	N	N	N	N	N
D7 24	N	N	N	N	N	N	N
E1 08	N	N	N	N	N	N	N
E1 13	N	N	N	N	N	N	N
**PATIENTS***							
**Sample (Nº)**	**Atopy**				**Medication**	**Comorbidities**	**Smoking status**
	**M**	**P**	**E**	**F**			
D4 28	N	Y	N	N	IC	Rhinitis	N
D4 38	N	Y	Y	N	IT	Rhinitis	N
D4 39	N	Y	N	N	IC	Rhinitis; Obesity	N
D4 41	N	Y	N	N	IT	Rhinitis	N
D4 49	N	Y	N	N	IC	Rhinitis	N
D4 50	N	Y	N	N	IC	Rhinitis	N
D4 52	N	Y	N	N	IT	Rhinitis	N
D4 54	N	Y	N	N	IC	Rhinitis	Y
D5 62	N	Y	N	N	IC	Rhinitis	N
D5 64	N	Y	N	N	IC	Rhinitis	N
D5 66	N	Y	N	N	IC; IT	Rhinitis	N
D5 76	N	Y	N	N	IT	Rhinitis	N
D7 06	N	Y	N	N	IC	Rhinitis	N
D7 15	N	Y	N	N	IC	Rhinitis	N
D7 17	N	Y	N	N	IC	Rhinitis	N
D7 81	N	Y	N	N	IC; SABA	Rhinitis; Polyposis	N
D8 18	N	Y	N	N	IC	Rhinitis	N
D8 32	N	Y	N	N	IC	Rhinitis	N
D8 67	N	Y	N	N	IT	Rhinitis; Celiac disease	N

E1 78	N	Y	N	N	IC; SABA	Rhinitis	N
E2 08	N	Y	N	N	ICt; IT	Rhinitis	N
E2 09	N	Y	N	N	IC	Rhinitis	N
E2 10	N	Y	N	N	IC	Rhinitis; Chron´s Disease	N
E2 11	N	Y	N	N	IC	Rhinitis	N
E2 13	N	Y	N	N	IC	n/a	N
E2 22	N	Y	N	N	IC	Rhinitis	N
E2 23	N	Y	N	N	IT	Rhinitis; Hypercholesterolemia	N
E2 25	N	Y	N	N	IC	Rhinitis	N
E2 29	N	Y	N	N	IC	Rhinitis	N
E2 34	N	Y	N	N	IC	Rhinitis	N

M: Mites; P: Pollen; E: Epithelium; F: Fungy; N: No; Y: Yes; IT: Immunotherapy; *All patients suffered asthma; IC: Inhaled Corticosteroid; SABA: Short-Acting Beta Agonist; n/a: not available.

Tabla S3. Multivariate logistic regression of the most differentially expressed genes in the transcriptomic analysis after adjustment with white blood cell counts.

**Up regulated genes**		**Logistic Regression (p-value)***					
**Ensemble ID**	**External ID Gene**	**Gene expression**	**Eosinophils**	**Neutrophils**	**Lymphocytes**	**Monocytes**	**Basophils**
ENSG00000161905	*ALOX15*	,151	,025	,402	,315	,762	,764
ENSG00000091181	*IL5RA*	,009	,096	,363	,142	,913	,901
ENSG00000103056	*SMPD3*	,029	,173	,578	,110	,686	,792
ENSG00000105205	*CLC*	,070	,038	,402	,195	,704	,922
**ENSG00000183134**	***PTGDR2***	**,037**	**,067**	**,426**	**,197**	**,820**	**,714**
ENSG00000134489	*HRH4*	,036	,027	,473	,383	,534	,614
ENSG00000152207	*CYSLTR2*	,003	,170	,586	,165	,769	,757
ENSG00000171659	*GPR34*	,013	,056	,932	,842	,379	,871
ENSG00000198502	*FCRL5*	,246	,001	,310	,201	,901	,829
ENSG00000143297	*RAB44*	,013	,079	,441	,151	,533	,686

**Down regulated genes**		**Logistic Regression (p-value)***					
**Ensemble ID**	**External ID Gene**	**Gene expression**	**Eosinophils**	**Neutrophils**	**Lymphocytes**	**Monocytes**	**Basophils**
ENSG00000118113	*MMP8*	,385	,004	,360	,372	,903	,849
ENSG00000012223	*LTF*	,422	,004	,350	,357	,961	,796
ENSG00000124469	*CEACAM8*	,404	,005	,331	,348	,973	,821
ENSG00000123689	*G0S2*	,702	,002	,355	,494	,704	,728
ENSG00000118520	*ARG1*	,197	,008	,371	,246	,929	,625
ENSG00000168209	*DDIT4*	,150	,002	,383	,719	,632	,786
ENSG00000179094	*PER1*	,042	,002	,324	,756	,678	,484
ENSG00000235169	*SMIM1*	,369	,003	,285	,345	,883	,922
ENSG00000255823	*MTRNR2L8*	,347	,002	,384	,402	,671	,780
ENSG00000096006	*CRISP3*	,161	,005	,374	,411	,969	,812

*p-value for **B** Parameter Estimate
